# Protective Mechanism of *Nostoc sphaeroides* Kütz. Polysaccharide on Liver Fibrosis by HFD-Induced Liver Fat Synthesis and Oxidative Stress

**DOI:** 10.1155/2022/1745244

**Published:** 2022-07-05

**Authors:** Litao Yang, Bo Zhang

**Affiliations:** Beijing Key Laboratory of Bioactive Substances and Functional Foods, Beijing Union University College of Biochemical Engineering, Beijing, China

## Abstract

*Nostoc sphaeroides* Kütz. polysaccharide (NSKP) is one of the main components of *Nostoc sphaeroides* Kütz. and is often used as health food. We investigated whether NSKP interferes with the progression of liver fibrosis. Male mice were randomly divided into 4 groups: control (C), high-fat diet (M), high-fat diet + 0.4 g/kg NSKP (L), and high-fat diet + 0.8 g/kg NSKP (H). C was fed standard diet, M was fed high-fat diet, and L and H were fed high-fat diet in addition to gavage of 0.4 g/kg or 0.8 g/kg NSKP, respectively, for 22 weeks. At the end of the experiment, the serum and liver oxidative stress, fat accumulation, and fibrosis indexes were detected. The histopathology of liver was also observed. The results showed that the rice of NSKP, compared with M, improved blood lipid level, liver total cholesterol (TC), triglyceride (TG), and liver antioxidant capacity and effectively interfered with liver fibrosis related indicators. So it is interesting to note that NSKP appeared to be effective in liver injury; further experiments are necessary to clarify the exact mechanisms involved.

## 1. Introduction

The excessive intake of high-fat and high-calorie foods increases the prevalence of hyperlipidemia, obesity, nonalcoholic fatty liver, type 2 diabetes, hypertension, coronary heart disease, and atherosclerotic disease [1, 2]. As an important organ of the human body, the liver plays an important role in metabolism, excretion, immunity, detoxification, and so on. Hyperlipidemia caused by dyslipidemia is an independent risk factor for chronic liver injury [3]. Chronic liver injury can initiate a vicious circle of hepatocyte apoptosis and death, inflammation, oxidative stress injury, and fibrosis and eventually lead to liver cirrhosis or hepatocellular carcinoma [4–6]. The early stage of lipid metabolic disorder caused by high-fat diet is mainly characterized by abnormal accumulation of fat; with continuous accumulation of fat, it may lead to oxidative damage of hepatocytes, lipid peroxidation, endoplasmic reticulum stress, mitochondrial dysfunction, hepatocyte apoptosis, or liver inflammation, thus leading to liver fibrosis to the degree of liver cirrhosis [7].

Hyperlipidemia is an important factor in liver fibrosis [8]. In addition, defects of ApoE genes lead to excessive production of potential lipids and lipoproteins, which disrupt lipid metabolism in the body and easily lead to hyperlipidemia and obesity [9]. Therefore, ApoE^−/−^ mice are often used to study lipid metabolism disorders and diseases caused by lipid metabolism disorders in various organs such as liver, kidney, artery, and brain nerves [10, 11]. Liver is the main site of lipid metabolism, and there is a close relationship between its injury and ApoE^−/−^ [12]. The occurrence of liver fibrosis is mainly due to the transformation of hepatic stellate cells to hepatic myofibroblasts, the proliferation of myofibroblasts, and the imbalance between the deposition and decomposition of extracellular matrix [13]. In the liver, hepatic stellate cells (HSC) are the main source of myofibroblasts that produce extracellular matrix; its main components are fibronectin (*FN*), collagen I (*Col-I*), and so on. Long-term liver injury will not only induce the transformation of hepatic stellate cells, but also cause a large amount of proliferation of transformed myofibroblasts [14, 15], and myofibroblasts are the main cells secreting extracellular matrix [16]. As one of the common symptoms of liver injury, hyperlipidemia can cause abnormal fat synthesis in the liver. A large amount of fat deposition in the liver can induce oxidative damage to hepatocytes, which triggers the secretion of transforming growth factor-*β*, a key factor for fibrosis, leading to the activation of hepatic stellate cells, characterized by the emergence of a large number of myofibroblasts expressing *α*‐smooth muscle actin (*α-SMA*) [17, 18]. Some studies have shown that the emergence of a large number of myofibroblasts causes the deposition of extracellular matrix and leads to liver fibrosis [19].

*Nostoc sphaeroides* Kütz. is a kind of lower unicellular cyanobacteria belonging to Cyanophyta, Cyanophyceae, Hormogonales, Nostocaceae, and *Nostoc*. It is commonly known as Nostoc sphaeroids or water fungus [20]. It is a kind of functional food for reducing fat, with anti-inflammation, anti-oxidation, and anti-atherosclerosis functions. It can be planted artificially, and it is a kind of economic cyanobacteria with high edible value [21, 22] and is rich in proteins, amino acids, fatty acids, vitamins, and other nutrients, of which the most important components are proteins and polysaccharides. Polysaccharides are important substances in *Nostoc sphaeroides* Kütz. [23]. A lot of studies have shown that plant polysaccharide extracts can protect the liver by improving the levels of lipids in serum and liver or anti-oxidation in the liver in animals with a high-fat diet [24]. Tang et al. have shown that the isolated polysaccharides from *Pueraria lobata* have the effect of scavenging free radicals [25]. Li Haifeng and others proved that NSKP has the function of anti-oxidation by using the *Caenorhabditis elegans* model [26]. Therefore, it is of great significance to study NSKP as a dietary supplement in the intervention of chronic diseases.

The main purpose of this study is to explore whether NSKP can interfere with liver fibrosis caused by long-term high-fat diet with improving oxidative stress caused by fat deposition in the liver of high-fat mice.

## 2. Materials and Methods

### 2.1. Materials

NSKP sample purity is 96.95% (provided by Hunan Yandi Bioengineering Co., Ltd., Hunan, China). The preparation method was as follows: 500 times the volume of distilled water was added to Kudzu rice powder that had been dried and crushed, extracted at 100°C for 2 hours, and then centrifuged at 9000 r/min for 15 min, and the supernatant was collected. After 10-time concentration, the supernatant was precipitated overnight in 40% (v/v) ethanol at 4°C and centrifuged at 9000 r/min for 15 min, and the supernatant and precipitate were collected and washed four times with 45% (v/v) ethanol. The precipitate was dissolved in distilled water and precipitated again in 40% (v/v) ethanol overnight at 4°C. After centrifugation at 9000 r/min for 15 min, the precipitate was collected, washed twice with 45% (v/v) ethanol, freeze-dried, and crushed into NSKP powder [23].

Assay kits used to measure total cholesterol (TC), triglyceride (TG), aspartate transaminase (AST), alanine transaminase (ALT), superoxide dismutase (SOD), glutathione (GSH), and malondialdehyde (MDA) were purchased from Nanjing Jiancheng Bioengineering Institute (Nanjing, China). Antibodies *GAPDH* (No. G3206-1OD), anti-transforming growth factor-beta 1 (*TGF-β1*, No. GB111876), *α-SMA* (No. GB13044), and anti-fibronectin (*FN*, No. GB11091); Sirius red staining kit; H&E staining kit; and oil red O staining kit were all provided by Wuhan Servicebio Co., Ltd. (Wuhan, China).

### 2.2. Animals and Experiment

The Animal Research Committee of Beijing Union University approved this study (license number: 201913). Eight-week-old male ApoE^−/−^ mice were purchased from Beijing Charles River Experimental Animal Technology Co., Ltd. (Beijing, SCXK (Jing) 2016–0006). The mice were placed in the SPF animal room of Beijing Union University and were freely provided with feed and drinking water in a 12-hour light-dark cycle at a constant temperature of 22°C ± 2°C.

After 40 ApoE^−/−^ mice were fed adaptively for one week with standard diet (SD), the mice were divided into 4 groups with 10 mice in each group. They were control group (C), high-fat diet (HFD) group (M), HFD + 0.4 g/kg BW group (L), and HFD + 0.8 g/kg BW group (H). During the 22-week study, the C was fed the SD, the M was fed the HFD, the L and H were fed HFD and given 0.4 g/kg BW or 0.8 g/kg BW NSKP, respectively, once a day, at 8 : 00 am, for 22 weeks. The SD and HFD were purchased from Medicience Ltd., Jiangsu, China (SD Product model: MD12014; HFD Product model: MD12015A; Certificate No. 2018–10030, Table 1). The experimental process is shown in Table 2, and the dose of NSKP was determined according to the previous experimental results.

Animals were weighed before and after experiment, and their food intake was measured. At the end of 22 weeks, all animals were fasted for 6 hours. At 6 am the next day, phenobarbital was injected intraperitoneally to anesthesia, and blood samples were collected. The animals were dissected, and the liver was quickly removed, weighed, and partially fixed in 4% paraformaldehyde phosphate buffer solution for histopathological examination. The rest of the liver was immediately placed in liquid nitrogen and stored at −80°C for later experiments.

### 2.3. Serum and Liver Indexes

After blood collection, the supernatant was centrifuged at 4°C, 3000 r/min, for 10 min to be used as serum, and AST and ALT were detected using kits.

The liver tissue frozen at −80°C was accurately weighed and mechanically homogenized in a ratio of weight (g) to homogenate medium (mL) of 1 : 9 under ice water bath. The homogenized tissue was prepared at 2500 r/min and centrifuged for 10 min. The supernatants were taken to detect the contents of TG, TC, AST, and ALT in liver tissue according to the corresponding kit. Protein concentrations were determined using the bicinchoninic acid (BCA) protein assay kit (Thermo Fisher Scientific). The data were normalized against the total protein level. Normal saline was used as homogenate to detect liver AST and ALT, and anhydrous ethanol was used as homogenate to detect liver TG and TC. Unless otherwise stated, the kits were purchased from Nanjing Jiancheng Institute of Biological Engineering.

### 2.4. Oxidative Stress Indicators

According to the ratio of weight (g) to normal saline (mL) of 1 : 9 and mechanical homogenate under ice water bath, the tissue homogenate prepared was 2500 r/min, centrifuged for 10 min. Taking supernatant and the appropriate dilution concentration was selected according to the corresponding kit for the detection of SOD, GSH, and MDA in liver according to the kit manufacturer's steps. Protein concentrations were determined using the bicinchoninic acid (BCA) protein assay kit (Thermo Fisher Scientific). The concentrations of SOD, GSH, and MDA in liver were calculated according to the data of total protein concentration.

### 2.5. Histopathology

The liver was fixed in 4% paraformaldehyde phosphate buffer solution to prepare 4 *μ*m cross-cut slices. Histopathological evaluation was performed by H&E staining, oil red O staining, and Sirius red staining. H&E staining was used to evaluate the morphology of liver tissue, oil red O staining was used to evaluate the lipid accumulation in liver tissue, and Sirius red staining was used to evaluate the degree of fibrosis in liver tissue. The Olympus D1027 microscope was used to randomly select 5 nonoverlapping fields, and Image-Pro Plus 6.0 graphic analysis software was used to calculate the percentage of positive area of fibrosis.

### 2.6. Immunohistochemical Staining

Liver fixed in 4% paraformaldehyde phosphate buffer solution was used to prepare 4 *μ*m cross-cut slices. The samples were randomly selected from each group. The *α-SMA* and *FN* of liver were determined according to the instructions of the immunohistochemical kit, and the immunohistochemical staining sections were quantified. The positive expression of brown-yellow or yellow particles was taken as positive expression. Five fields were randomly selected from each section, and the average optical density of positive staining area was detected by Image-Pro Plus 6.0 analysis software (Area Optical Density = IOD/Area).

### 2.7. Western Blotting (WB)

The liver at −80°C was used. The cytokine *TGF-β1* (with the dilution ratio of antibody concentration being 1 : 1000) in liver was detected, and *β*-actin was used as a normalize control for protein loading. Total protein was extracted from liver samples by homogenization with RIPA extraction buffer (Servicebio, Wuhan, China). The protein concentration of liver samples was measured using a BCA method (Nanjing Jiancheng Bioengineering Institute, Nanjing, China). Protein samples were then subjected to 10% sodium dodecyl sulfate–polyacrylamide gel electrophoresis separation (SDS-PAGE) and transferred to nitrocellulose membranes. Membranes were blocked for 1 h using 5% bovine serum albumin (BSA)/phosphate buffered saline (PBS), after which they were probed overnight with appropriate primary antibodies at 4°C. Blots were then washed using PBS and probed using appropriate horseradish peroxidase-linked secondary antibodies. The membrane bands were displayed by chemiluminescence (micropores), and the strip intensity of each channel was quantified by ImageJ 6.0 software.

### 2.8. Reverse Transcription-Polymerase Chain Reaction (RT-PCR)

Liver tissue cryopreserved at −80°C was used. TRIzol was used to extract total RNA from mouse liver, and the concentration and purity of RNA were measured by nucleic acid protein analyzer at 260/280 nm. Then, the cDNA was reverse-transcribed by Fast Pfus PCR Master Mix and then operated according to the kit. The primers *GAPDH*, sterol regulatory element binding transcription factor 1 (*SREBP-1c*), fatty acid synthesis (*FAS*), *TGF-β1*, *α*-smooth muscle actin (*α-SMA*), fibronectin (*FN*), and collagen I (*Col-I*) were designed by DNAMAN software. The primers were synthesized by Wuhan Sewell Biotechnology Co., Ltd., and detected by Bio-Rad CFX96™ fluorescence quantitative PCR system. PCR procedure was as follows: 95°C for 2 min; 95°C for 20 s, 55°C for 20 s, 72°C for 20 s, 30 cycles; 72°C for 10 s. Using *GAPDH* as internal reference, the relative expression levels of the genes to be measured were expressed as 2^−ΔΔCt^. ^ΔΔ^ Ct = [Ct target gene (sample to be tested) − reference within Ct (sample to be tested)] − [Ct target gene (calibration sample) − reference within Ct (calibration sample)]. Primer sequences are shown in Table 3.

### 2.9. Statistical Analysis

Statistical analysis was conducted using SPSS software for Windows (version 22). Data were assessed using one-way ANOVA and Newman–Keuls pairwise comparison. *P* < 0.05 values were considered significant differences. All data from these assays are shown as mean ± SEM.

## 3. Results

### 3.1. Effects of NSKP on Body Weight and Food Intake

As shown in Table 4, compared with group M, groups L and H had no significant differences in the final body weight and total food intake (*P* > 0.05), indicating that NSKP had no effect on the body weight and total food intake of high-fat diet mice.

### 3.2. Effects of NSKP on Lipid and Transaminase in the Serum and Liver of High-Fat Diet Mice

**Figure 1:**
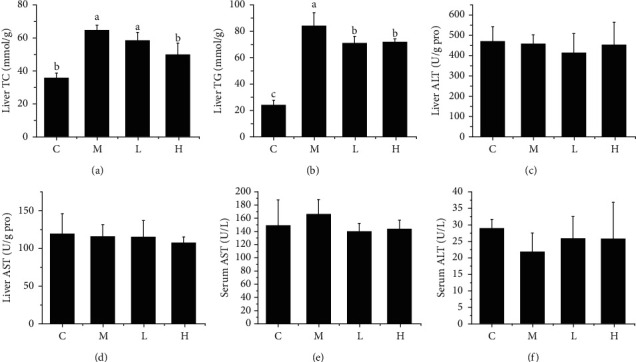
Effects of NSKP on serum and liver indexes of high-fat mice. (a) Liver TC. (b) Liver TG. (c) Liver ALT. (d) Liver AST. (e) Serum ALT. (f) Serum AST. Bars marked with different letters represent statistically significant (*P* < 0.05), whereas bars labeled with the same letter indicate no statistically significant difference between the groups (*P* > 0.05). Values represent mean ± SEM; *n* = 10 in each group.

Lipids and transaminases in serum and liver were detected at the end of 22 weeks. Our previous study has concluded that NSKP significantly reduced serum TC, TG, and LDL-C and increased HDL-C content in mice fed a high-fat diet for 22 weeks (title: Effects of *Nostoc sphaeroides* Kütz. Polysaccharide on Renal Fibrosis in High-Fat Mice, accepted). As shown in Figures 1(a) and 1(b), liver TC and TG in group M were significantly increased compared with group C, indicating that 22-week high-fat diet led to liver lipid metabolism disorder, but gavage of different doses of NSKP (L and H) significantly reduced liver TC and TG contents, respectively. There was no significant difference in ALT and AST in serum and liver (Figures 1(c)–1(f)) among all groups (*P* > 0.05). The results showed that NSKP ameliorated lipid disorder in high-fat diet mice.

### 3.3. Effects of NSKP on Liver Tissue Structure

**Figure 2:**
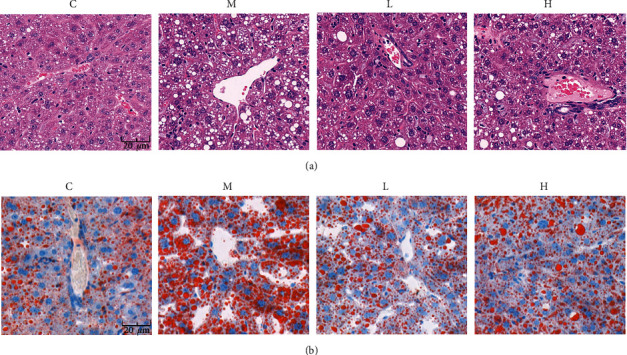
Effects of NSKP on liver of high-fat mice. (a) H&E staining in the liver. (b) Oil red O staining in the liver. Original magnification: 400x.

Histopathology can intuitively observe the morphology and structure of tissues. According to H&E staining in Figure 2(a), liver vacuolation was severe in group M compared with group C, the structure of middle liver cells was unclear and showed swelling and necrosis, and the arrangement of cells was disordered. However, the vacuolation was relatively reduced in groups L and H, and the cell structure was clearly visible after NSKP treatment. According to oil red O staining in Figure 2(b), compared with group C, group M showed increased red lipids in liver and serious lipid accumulation, but the red area and lipid accumulation were reduced in group L and H after being fed with NSKP. The results showed that NSKP improved liver injury and steatosis of high-fat diet mice.

### 3.4. Effects of NSKP on mRNA of Liver Fat Synthesis

**Figure 3:**
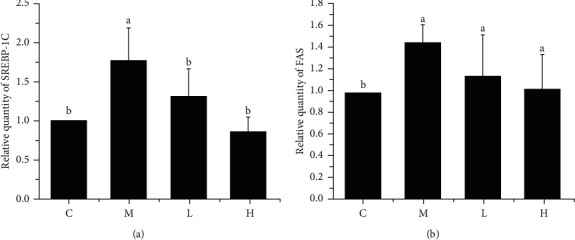
Effects of NSKP on mRNA of fat. (a) Relative quantity of *SREBP-1c* by RT-PCR. (b) Relative quantity of *FAS* by RT-PCR. Bars marked with different letters represent statistically significant (*P* < 0.05), whereas bars labeled with the same letter indicate no statistically significant difference between the groups (*P* > 0.05). Values represent mean ± SEM; *n* = 10 in each group (*SREBP-1c*: sterol regulatory element binding transcription factor 1-c, *FAS*: fatty acid synthesis).

Fat synthesis genes play a key role in fat synthesis. We detected the key fat synthesis genes in the liver by RT-PCR technology. As shown in Figure 3(a), compared with group C, group M showed significantly increased mRNA expression of *SREBP-1c*, but the mRNA expression of *SREBP-1c* in groups L and H was significantly decreased after gavage of NSKP (*P* < 0.05, *P* < 0.05). As shown in Figure 3(b), the mRNA expression of *FAS* in group M was significantly increased compared with group C (*P* < 0.05), indicating that *FAS* in liver of mice was increased lipid disorders, and there was a trend of decrease after gavage of NSKP, but there was no significant difference (*P* > 0.05). The results showed that NSKP reduced the key mRNA of fat synthesis in high-fat diet mice.

### 3.5. Effects of NSKP on Liver Oxidative Stress

**Figure 4:**
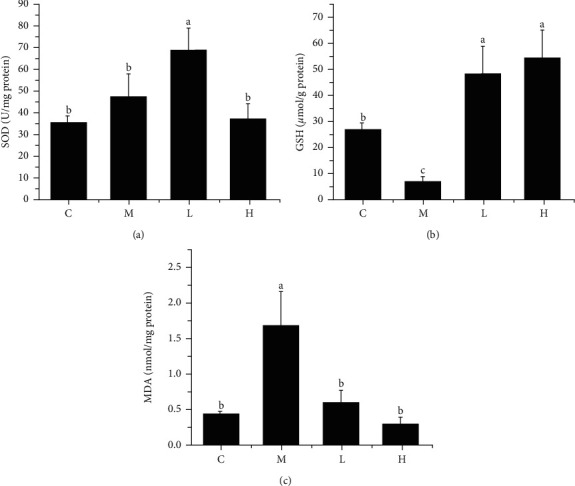
Effects of NSKP on liver oxidative stress in high-fat mice. (a) SOD (U/mg protein). (b) GSH (*μ*mol/g protein). (c) MDA (nmol/mg protein). Bars marked with different letters represent statistically significant (*P* < 0.05), whereas bars labeled with the same letter indicate no statistically significant difference between the groups (*P* > 0.05). Values represent mean ± SEM; *n* = 10 in each group (SOD: superoxide dismutase, GSH: glutathione, MDA: malondialdehyde).

Long-term high-fat diet can cause oxidative stress in mice liver. Based on the phenomenon of oxidative stress caused by dyslipidemia, we used corresponding kits to detect the indicators of oxidative stress in mice liver. It can be seen from Figure 4(a) that there was no significant difference in SOD content between group M and group C, but liver SOD contents (L) of high-fat mice fed with NSKP were significantly increased (*P* > 0.05, *P* < 0.05). As can be seen from Figures 4(b) and 4(c), liver GSH of mice was significantly decreased and liver MDA increased (*P* < 0.05, *P* < 0.05) after being fed with high-fat diet, but liver GSH of mice was significantly increased and liver MDA decreased (*P* < 0.05, *P* < 0.05) after gavage of NSKP. The results showed that NSKP improved the antioxidant capacity in liver of high-fat diet mice.

### 3.6. Effects of NSKP on *TGF-β1*, *α-SMA*, and *FN* in the Liver

**Figure 5:**
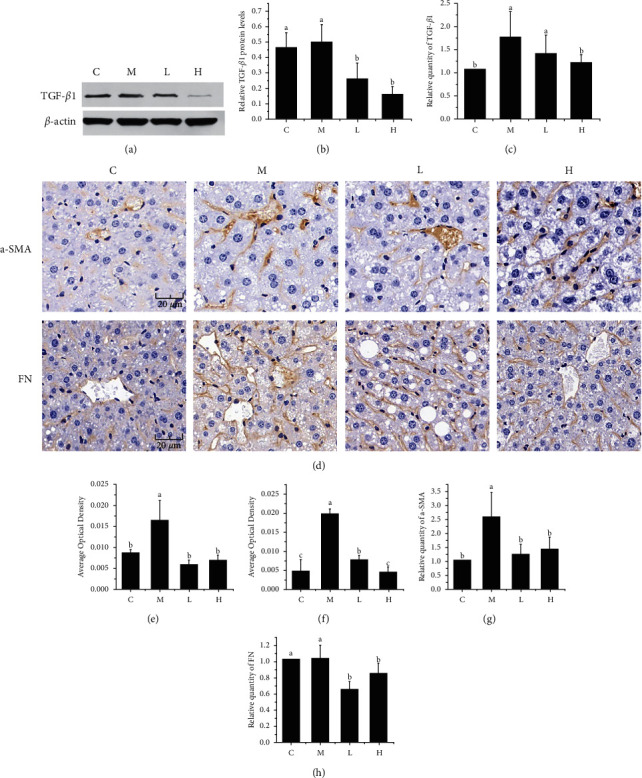
Effects of NSKP on hepatic *TGF-β1*, *α-SMA*, and *FN* in high-fat mice. (a) The blot of *TGF-β1* by WB. (b) Protein expression of *TGF-β1*. (c) Relative quantity of *TGF-β1* by RT-PCR. (d) Immunohistochemical staining (*α-SMA*, *FN*), original magnification: 400x. (e) Quantitative analysis of the average optical density of *α-SMA* immunohistochemical staining sections. (f) Quantitative analysis of the average optical density of *FN* immunohistochemical staining sections. (g, h) Relative quantity of *α-SMA* and *FN* by RT-PCR. Bars marked with different letters represent statistically significant (*P* < 0.05), whereas bars labeled with the same letter indicate no statistically significant difference between the groups (*P* > 0.05). Values represent mean ± SEM; *n* = 10 in each group (*TGF-β1*: transforming growth factor-beta 1; *α-SMA*: *α*-smooth muscle actin; *FN*: fibronectin).

*TGF-β1*, *α-SMA*, and *FN* are important markers of fibrosis, which seriously affect the development of liver fibrosis. As can be seen from Figures 5(a)–5(c), the protein expression of *TGF-β1* in group M was not significantly different compared with group C (*P* > 0.05), but the mRNA expression of *TGF-β1* was significantly increased (*P* < 0.05), and the protein expression and mRNA expression of *TGF-β1* were significantly decreased (*P* < 0.05) after feeding mice with NSKP. The results showed that NSKP decreased the expression of *TGF-β1* in liver of high-fat diet mice.

As shown in Figures 5(d)–5(f), immunohistochemical staining of *α-SMA* and *FN* showed that the positive area of *α-SMA* and *FN* in group M was increased and Area Optical Density was significantly increased compared with group C (*P* < 0.05, *P* < 0.05), but after the intervention of NSKP, the positive area of *α-SMA* and *FN* was decreased. Area Optical Density was significantly decreased (*P* < 0.05, *P* < 0.05). RT-PCR assays were performed on *α-SMA* and *FN*. As shown in Figures 5(g) and 5(h), the mRNA expressions of *α-SMA* and *FN* were significantly decreased after 22 weeks of NSKP intervention (*P* < 0.05, *P* < 0.05). The results indicated that NSKP reduced the expression of *α-SMA* and *FN* in liver of high-fat diet mice.

### 3.7. Effects of NSKP on Liver Fibrosis

**Figure 6:**
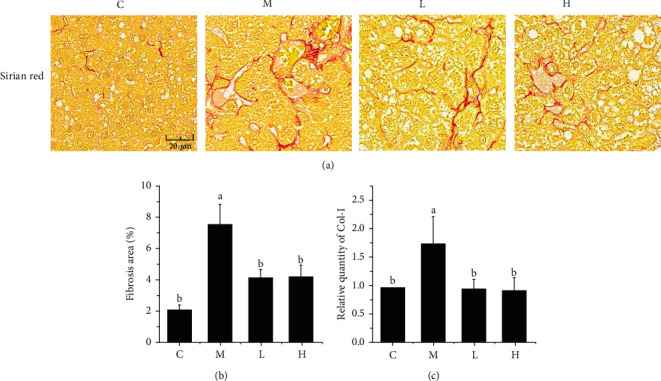
Effect of NSKP on hepatic collagen fibers in high-fat mice. (a) Sirius red staining of the liver; red represents collagen fibers; original magnification: 400x. (b) Fibrosis area (%). (c) The mRNA expression levels of *Col-I* were detected by RT-PCR. Bars marked with different letters represent statistically significant (*P* < 0.05), whereas bars labeled with the same letter indicate no statistically significant difference between the groups (*P* > 0.05). Values represent mean ± SEM; *n* = 10 in each group (*Col-I*: collagen I).

The main cause of liver fibrosis is the accumulation of fibrin in the hepatic Disse space. According to Figures 6(a) and 6(b), compared with group C, group M showed significantly increased red part and area of fibrosis (*P* < 0.05). After 22 weeks of NSKP treatment, the red part was significantly decreased and the area of fibrosis was significantly decreased (*P* < 0.05). As shown in Figure 6(c), the mRNA expression of *Col-I* was significantly decreased in high-fat diet mice after 22 weeks of NSKP treatment (*P* < 0.05). The results showed that NSKP ameliorated liver fibrosis in high-fat diet mice.

## 4. Discussion and Conclusion

Liver fibrosis is the persistent result of chronic liver injury, that is, an abnormal healing reaction caused by liver injury, which is a reversible process, but if not treated in time, it will cause liver cirrhosis and eventually lead to liver failure [27]. High-fat diet can cause dyslipidemia, and dyslipidemia is another important risk factor for liver fibrosis in addition to obesity and type 2 diabetes [28]. A long-term high-fat diet can also induce chronic liver diseases such as hepatitis and liver fibrosis. Studies have shown that it can cause abnormal synthesis and accumulation of fat in the liver and induce oxidative stress and inflammatory reactions. Then, it stimulates the fiber to form a signal pathway and cause liver fibrosis [29].

As a functional food of natural biological origin, *Nostoc sphaeroides* Kütz. is a kind of single-celled blue algae and has a good lipid-lowering effect, which can directly reduce the absorption of cholesterol in the intestinal tract and inhibit atherosclerotic lesions without increasing the burden of liver metabolism [21]. Previous studies in our laboratory have proved that *Nostoc sphaeroides* Kütz. can inhibit liver injury and improve intestinal microorganisms [22, 30]. The content of polysaccharides in *Nostoc sphaeroides* Kütz. is about 40%. The monosaccharide composition of NSKP connects arabinose, galactose, glucose, xylose, mannose, and glucuronic acid through *β*-1-3 glycosidic bond and *β*-1-4 glycosidic bond. It is an important substance in *Nostoc sphaeroides* Kütz. [26]. Some studies have shown that the important mechanism of polysaccharides extracted from *Lycium barbarum* in improving liver fibrosis induced by high-fat diet may be the improvement of oxidative stress in the liver [31]. Phaeophyceae extract can reduce liver fibrosis in high-fat male C57BL/6J mice by intervening in abnormal oxidative stress and inflammatory response induced by high-fat diet [32]. Therefore, this experiment mainly studies the effect of NSKP on liver lipid content and oxidative stress in hyperlipidemic mice, which may achieve the effect of preventing liver fibrosis.

Hepatic steatosis is caused by increased de novo fat synthesis rate and decreased fat oxidation rate [33, 34]. Two important factors of fat accumulation in the liver are blocked free fatty acid transport and abnormal increase of fat synthesis, and the genes *SREBP-1c* and *FAS* of fat synthesis play a key role in steatosis [35, 36]. It has been reported that *SREBP-1c* can regulate *FAS* expression and lead to TG synthesis, and the mechanisms underlying the effects of *SREBP-1c* on hepatic stellate cell activation and liver fibrosis were involved in its influences on the receptors for *TGF-β1* and PDGF-*β* and their downstream signaling, and the molecules for epigenetic regulation of genes [37]. The high-fat diet has been confirmed to cause TG and TC accumulation by increasing *SREBP-1c* and *FAS* expression [38, 39]. A large number of studies have shown that plant extracts can reduce liver steatosis by improving key genes of liver fat synthesis [40, 41]. In our experiment, NSKP reduced the lipid level of high-fat diet mice fed for 22 weeks. After histopathological staining sections, it was observed that the liver structure of high-fat mice had abnormal damage and the fat accumulation was serious. On the contrary, after NSKP feed, liver injury was alleviated and fat accumulation was improved. At the same time, the mRNA expression of *SREBP-1c* and *FAS* was decreased, suggesting that NSKP interfered with the accumulation of fat in liver by reducing *SREBP-1c* and *FAS*, which are important genes in liver fat synthesis.

The increase of liver fat load and the occurrence of lipotoxicity can lead to a high level of oxidative stress in animal models of liver fat with fibrosis, mainly due to the reduction of antioxidant level [42, 43]. According to the double-hit hypothesis, the aggravation of fat accumulation in liver cells will lead to oxidative damage of liver cells, which is one of the key mechanisms in liver fibrosis. The decrease of antioxidant secretion and the production of lipid peroxides both accelerate the damage of liver cells and the abnormal phenomena of liver tissue structure [7]. The experimental results showed that SOD increased significantly and MDA decreased significantly after NSKP feed, indicating that the antioxidant capacity of liver tissue was gradually increased and the production of lipid peroxides was decreased. Further, it was shown that NSKP alleviated the phenomenon of oxidative stress in liver tissue caused by long-term high-fat diet. Thus, the abnormal liver tissue structure and lipid accumulation were improved.

Hepatocytes can participate in the activation of hepatic stellate cells through various mechanisms, such as the stress response or inflammatory response, thus affecting liver fibrosis [44]. Studies have shown that the activation of hepatic stellate cells is generated by direct interaction with the stress or death of hepatic cells [45]. Proliferation of hepatic stellate cells is also stimulated by growth factors, such as transforming growth factor (TGF) and epidermal growth factor [46, 47]. At the same time, growth factors promote the remodeling of the extracellular matrix (ECM), leading to the formation of collagen fibrin. In normal liver, collagen IV and collagen VI are present in the Disse space of the liver. However, they are replaced by type I and II collagen and fibronectin during fiber formation [48]. *TGF-β1* is a key signal molecule of renal fibrosis and the strongest key driver of liver fibrosis [18, 49]. It is normally inactive, but when activated, it activates the signaling pathway through Smad protein to stimulate the development of fibrosis mechanism and lead to the production of collagen fibrin [50, 51]. In addition, *TGF-β1* promotes the transdifferentiation of ECM-secreting myofibroblasts [52]. The stress response of the liver caused by long-term lipid metabolism disorders leads to the massive production of *TGF-β1*, which further stimulates the activation of hepatic stellate cells. The most obvious phenomenon is the excessive secretion of *α-SMA*, which is also an indispensable key marker in the development of liver fibrosis [53]. The presence of *TGF-β1* also seriously affects the degradation mechanism of the ECM (mainly *Col-I* and *FN*), leading to excessive deposition of extracellular matrix in the liver Disse space, inducing the damage of liver tissue structure, and ultimately leading to liver fibrosis [54, 55]. It is worth noting that in this experiment, *TGF-β1* and *α-SMA* in the liver of high-fat mice were reduced to varying degrees after gavage of NSKP, indicating that NSKP regulated *TGF-β1* and *α-SMA* key cytokines in liver fibrosis of high-fat mice, so as to achieve the effect of liver fibrosis intervention. According to histopathological staining section observation of the results of this experiment, the liver tissue structure of HFD group mice fed high fat diet for a long time had abnormal changes, with serious collagen fiber deposition and severe fibrosis in liver. The expression of FN and type I collagen fibrin in mice fed high fat diet was downregulated after given NSKP. It slowed down the deposition of extracellular matrix in liver of high fat diet mice and also improved liver fibrosis.

In conclusion, the intervention of NSKP improved liver steatosis and oxidative stress and delayed the progression of liver fibrosis in mice on a long-term high-fat diet.

## Figures and Tables

**Table 1:** The ingredient of the diet (g/100 g).

Ingredient	Standard diet	High-fat diet
Cornstarch	20.96	5.00
Casein	19.47	19.47
Maltodextrin	9.98	9.98
Anhydrous milk fat	3.97	19.72
Corn oil	1.28	0.99
Sucrose	34.00	34.00
Fiber	4.99	4.99
Mineral mix	3.88	3.88
Vitamin mix	1.47	1.47
Cholesterol	0	0.5

**Table 2:** Procedure of experiment.

	Diet	ApoE^−/−^			
0^th^ week-1^th^ week	SD	√			
	HFD	—			
		C	M	L	H
2^nd^ week –22^nd^ week	SD	√	—	—	—
	HFD	—	√	√	√
	0.4 g/kg NSKP	—	—	√	—
	0.8 g/kg NSKP	—	—	—	√

*Note*. C: SD; M: HFD; L: HFD + 0.4 g/kg NSKP; H: HFD + 0.8 g/kg NSKP.

**Table 3:** Primers used for RT-PCR

Accession ID	Genes	Forward (5′ to 3′)	Reverse (5′ to 3′)
NM_008084.2	*GAPDH*	CCTCGTCCCGTAGACAAAATG	TGAGGTCAATGAAGGGGTCGT
NM_001313979.1	*SREBP-1c*	TCTGTGAGAAGGCCAGTGGGTA	GAGCTGTGGCCTCATGTAGGAATA
NM_007987.2	*FAS*	TAGAACCTCCAGTCGTGAAACCA	ATCTCATCTATCTTGCCCTCCTTG
NM_011577.2	*TGF-β1*	TAATGGTGGACCGCAACAAC	CCACATGTTGCTCCACACTTGAT
NM_007392.3	*α-SMA*	GTACCACCATGTACCCAGGC	GAAGGTAGACAGCGAAGCCA
NM_001276408.1	*FN*	ACACGGTTTCCCATTACGCC	GGTCTTCCCATCGTCATAGCAC
NM_007742.4	*Col-I*	GAGAGGTGAACAAGGTCCCG	AAACCTCTCTCGCCTCTTGC

**Table 4:** Effects of NSKP on body weight and food intake of high-fat mice.

	Body weight (g)		Total food intake (g)
	0^th^ week	22^nd^ week	
*C*	23.26 ± 0.78	29.79 ± 1.52	515.73 ± 31.91^a^
*M*	23.05 ± 0.46	30.06 ± 1.81	463.51 ± 37.23^ab^
*L*	23.19 ± 0.72	31.76 ± 1.78	444.34 ± 26.27^b^
*H*	22.72 ± 0.55	32.79 ± 2.07	456.65 ± 18.53^ab^

Values represent mean ± SEM; *n* = 10 in each group.

## Data Availability

The data used to support the findings of this study are included within the article.
